# Effects of lifestyle on telomere length: A study on the Korean population

**DOI:** 10.1371/journal.pone.0325233

**Published:** 2025-06-25

**Authors:** Chul-young Bae, In-hee Kim, Sun-Hyun Kim, Hyejin Chun, Bo-seon Kim, Min-hee Jeon

**Affiliations:** 1 Department of Mediage Research Center, Seongnam-si, Gyeonggi-do, Republic of Korea; 2 Moadata, Seongnam-si, Gyeonggi-do, Republic of Korea; 3 Department of Family Medicine, International St. Mary’s Hospital College of Medicine, Catholic Kwandong University, Incheon, Republic of Korea; 4 Ewha Womans University College of Medicine, Seoul, Republic of Korea; International Medical University, MALAYSIA

## Abstract

Telomere length is a known indicator of biological aging, typically decreasing with age. Biological age is a benchmark for assessing an individual’s health and aging. Correlations between telomere length and lifestyle factors have primarily been investigated from the perspective of a single variable and predominantly examined in postmenopausal women in Korea. This study aimed to analyze the effects of multiple lifestyle factors on telomere length in a diverse Korean population comprising 368 healthy adults (174 men and 194 women). We measured anthropometric and blood-related parameters and collected data on lifestyle-related factors, such as exercise, smoking, alcohol consumption, sleep, and stress, using surveys. Telomere length was quantified using monochrome multiplex quantitative real-time polymerase chain reaction (qPCR), and the relationship between lifestyle factors and telomere length was analyzed using correlation and regression analyses (*p*-value <0.10). Our findings indicated that telomere age, derived from telomere length, significantly increased with each adverse lifestyle factor. For men, significant contributors included exercise, smoking, and stress, whereas for women, significant contributors were exercise, alcohol consumption, sleep, and stress. The results showed that lifestyle and biological age considerably affected telomere age and accelerated the aging process. These results emphasize the importance of lifestyle in the management of biological aging.

## Introduction

Telomeres are hat-shaped nucleoprotein structures at the ends of eukaryotic chromosomes. They play a crucial role in protecting chromosomes against oxidative stress and other harmful substances. Although telomeres lack genetic information as chromosomal structures, they have recently been identified as important biomarkers of aging [1–3].

As cells divide, telomeres gradually shorten. However, the rate of this shortening varies depending on factors, such as sex, age, and stress, the presence of diseases, such as diabetes or hypertension, and lifestyle-related habits, such as exercise, alcohol consumption, and smoking [4–11]. Reduced telomere length (TL) impairs tissue regeneration and stem cell differentiation, causes abnormalities in tissue or cellular metabolic functions, increases the levels of harmful inflammatory compounds associated with cellular aging, and increases the risk of tumorigenesis due to genetic instability. Several studies have found that TL is shorter in individuals with cardiovascular disease, atherosclerosis, hypertension, diabetes, and cancer than that in healthy individuals [12,13]. Numerous studies have provided significant insights into the role of telomeres in health and disease, and simple techniques have been developed to measure telomere-related variables. The relationship between telomeres and various diseases is an ongoing research area [14–20]. However, telomere length has emerged as a biomarker for assessing cellular health, aging, and aging management. In line with this, telomere length measurements are often used to estimate biological age (BA), which reflects an individual’s general health status and aging process [21].

“Age” generally denotes chronological age (CA) and is typically used to assess overall health and aging; however, it is merely a numerical representation of time and cannot objectively evaluate physiological functions or health states. In contrast, BA reflects overall physiological functions, acknowledging that even individuals born in the same year can age at different rates [22–33]. Various studies have shown that BA can comprehensively analyze health encompassing biochemical, functional, and physiological aspects, thus offering a more objective assessment of individual health [22–24,26,34–36].

Several studies have indicated a correlation between TL and lifestyle-related factors; however, most of these studies have focused on individual factors. Telomere length is also related to racial characteristics [37,38], but studies in Korea have predominantly focused on postmenopausal women [39–43]. Consequently, there is a need to investigate the correlations between TL and BA in a broader Korean population. To address this issue, this study investigated the effects of multiple lifestyle-related factors on TL in male and female Korean adults.

## Methods

### Study participants

This study was approved by the Institutional Review Board of Catholic Kwandong University International St. Mary’s Hospital (CKU ISH; IS15TIMI0005). This study was conducted solely for academic purposes and involved voluntary written consent from healthy individuals who visited for a health examination. Participants were recruited from March 2015 to May 2015, and blood samples were collected at the CKU ISH Health Examination Center. In total, 368 healthy Korean adults (aged 23–90 years) were selected based on the established inclusion and exclusion criteria. The minimum number of participants required for this study was 274 (beta 0.04, power 80%, and probability 0.05). The total number of recruits (368) provided a statistically significant sample size. Personal information was collected, including participants’ names, dates of birth, ages, and examination dates. All personal information was encrypted and stored; participant identification information was inaccessible. Data on age, sex, and lifestyle-related factors, including smoking, alcohol consumption, and stress levels, were collected using a survey.

### Inclusion criteria

The inclusion criteria for this study were as follows:

1) An adult aged 20 years or older.
2) An individual willing to participate and voluntarily sign a written consent.

### Exclusion criteria

The exclusion criteria for patients in this study were as follows:

1) Non-Korean or individuals from ambiguous ethnic backgrounds.
2) Pregnant or lactating women.
3) Patients with severe illness (defined as having a serum creatinine level 1.5-fold higher than the normal upper limit).
4) Patients with severe liver conditions, such as cirrhosis (defined as having aspartate aminotransferase [AST] or alanine aminotransferase [ALT] levels 3-fold higher than the normal upper limit).
5) Patients with autoimmune diseases (such as multiple sclerosis, lupus, and rheumatoid arthritis).
6) Patients with uncontrolled diabetes (hemoglobin A1c (HbA1c) ≥8.0%).
7) Patients with diabetes undergoing treatment with sulfonylureas or insulin.
8) Patients with uncontrolled hypertension (systolic blood pressure [SBP] ≥150 mmHg or diastolic blood pressure [DBP] ≥100 mmHg).
9) Patients with uncontrolled hyperlipidemia (low-density lipoprotein-cholesterol [LDL-C] ≥160 mg/dL).
10) Patients with uncontrolled thyroid dysfunction.
11) Patients with dementia or a psychiatric disorder.
12) Individuals who received a systemic steroid injection during the 2 weeks before their hospital visit.
13) Individuals with a psychiatric illness or insomnia who received antipsychotic drugs for two or more weeks before their visit.
14) Individuals who took herbal medicine during the 2 weeks before their hospital visit.
15) Individuals who participated in another clinical trial involving test drugs or foods during the 2 weeks before their hospital visit.
16) Individuals who took health functional foods (excluding vitamins or minerals) during the 2 weeks before their hospital visit.
17) Patients with other clinically significant medical conditions (e.g., neurological or psychiatric diseases, cardiovascular diseases, gastrointestinal diseases, and malignant tumors) that may affect test results or patients determined ineligible for the study.

### TL measurement

Genomic DNA was extracted from peripheral blood leukocytes using a QIAamp DNA Blood Mini Kit (Qiagen, Valencia, CA, USA), according to the manufacturer’s instructions. All DNA samples were diluted to equal concentrations based on UV absorbance and stored at −80 °C. Using monochrome multiplex quantitative real-time polymerase chain reaction (MMQPCR) [42], TL was measured as the number of repeated copies for the telomere/single copy gene ratio (TSR). Real-time PCR was performed using the Quantstudio^TM^ 6 Flex Real-Time PCR System (Applied Biosystems, Waltham, MA, USA). The accumulation rate of the amplified DNA was continuously monitored at a final concentration of 0.0625 U using Power SYBR Green Master Mix (Applied Biosystems) and Titanium Taq DNA polymerase (Clontech, Mountain View, CA, USA). The primers used for telomere PCR were 5′-TGT TAG GTA TCC CTA TCC CTA TCC CTA TCC CTA TCC CTA ACA-3′ and 5′-ACA CTA AGG TTT GGG TTT GGG TTT GGG TTT GGG TTA GTG T-3′ (both at 400 nmol/L each). The primers used for the beta-globin (single copy gene) PCR were 5′-CGG CGG CGG GCG GCG CGG 7AGT CT CGG CTT CAT CCA CGT TCA CCT TG-3′ and 5′-GCC CGG CCC GCC GCG CCC GTC CCG CCG GAG GAG AAG TCT GCC GTT-3′ (both at 400 nmol/L each). An identical baseline threshold was applied to all wells of each MMQPCR plate regarding the amplified curves of both telomere and single-copy gene signals. Standard DNA was used, spanning 27-fold serial dilutions (60–2.22 ng). Triplicates of two standard samples with TSR values of 0.5 or 1.0 were included in all MMQPCR plates.

### Variables

#### Biomarkers.

In total, 31 clinical markers were collected, including 10 anthropometric and 21 blood parameters. The anthropometric parameters included height (HT), weight (WT), body fat (BF), lean body mass (LBM), waist circumference (WC), hip circumference (HC), SBP, DBP, forced expiratory volume in 1 s (FEV1), and forced vital capacity (FVC). The blood parameters included total protein (TP), albumin (ALB), total bilirubin (TB), AST, ALT, gamma GTP (GGT), total cholesterol (TC), triglyceride (TG), high-density lipoprotein-cholesterol (HDL-C), LDL-C, homocysteine (HOMO), alkaline phosphatase (ALP), lactate dehydrogenase (LDH), creatine phosphokinase (CPK), fasting blood sugar (FBS), HbA1c, blood urea nitrogen (BUN), creatinine (CR), red blood cell (RBC), hemoglobin (HEMO), and hematocrit (HEMA). BA was subsequently calculated from these clinical markers using the MEDIAGE^TM^ Biological Age Measurement System (MEDIAGE, Gyeonggi-do, South Korea).

#### Lifestyle-related parameters.

Lifestyle-related parameters included exercise, smoking, alcohol consumption, sleep, and stress. Exercise and sleep were categorical variables, whereas alcohol consumption, smoking, and stress were continuous variables. Sleep duration was categorized into three groups, with 7–8 h being the optimal duration for adults according to previous studies [44–46]. Smoking history was calculated using pack-years (PACK_YEAR), which was calculated according to the criteria set by the National Cancer Institute (NCI) [47]. For alcohol consumption, weekly intake (WEEK_ALCOHOL) was calculated by multiplying the frequency of alcohol consumption per week by the average number of glasses consumed per occasion based on daily alcohol consumption [48,49]. The PACK_YEAR and WEEK_ALCOHOL calculations are shown in Eq. 1 and 2.


$$\begin{array}{c} \text{PACK\textbackslash{}\_ YEAR }=\;(\text{number\textbackslash{} of\textbackslash{} cigarettes\textbackslash{} smoked\textbackslash{} per\textbackslash{} day}/\;20)\;\times \text{ number\textbackslash{} of\textbackslash{} smoking\textbackslash{} years} \end{array}$$ (1)

$$\begin{array}{c} \begin{array}{c} \text{WEEK\textbackslash{}\_ ALCOHOL }=\;(\text{weekly\textbackslash{} alcohol\textbackslash{} intake }\times \text{ average\textbackslash{} number\textbackslash{} of\textbackslash{} glasses\textbackslash{} per\textbackslash{} serving }\\ \times \text{ amount\textbackslash{} of\textbackslash{} alcohol\textbackslash{} consumed\textbackslash{} per\textbackslash{} serving\textbackslash{} (50\textbackslash{} mL) }\\ \times \text{ alcohol\textbackslash{} content }(22\%)\;\times \text{ specific\textbackslash{} gravity\textbackslash{} of\textbackslash{} alcohol }(0.8\;g))/\;100 \end{array} \end{array}$$ (2)

This study analyzed the effects of lifestyle factors on telomere age (TMA) based on survey results.

#### Telomere-related parameters.

Telomere-related parameters included TSR and TL. MMQPCR [50] was used to measure TL. This study applied TMA, a BA model based on TL, as the final variable for analysis.

*1) TL estimation and standardization*

**Fig 1:**
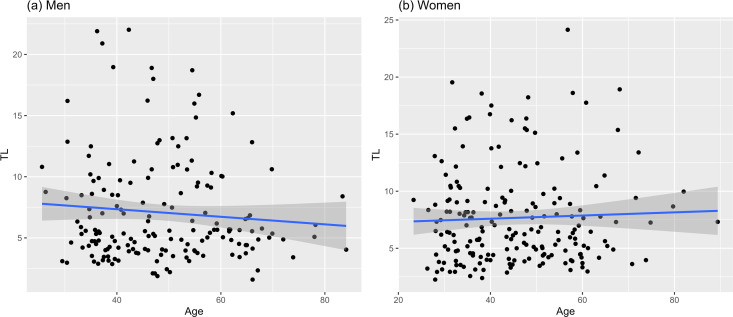
Correlation between age and telomere length (TL) according to sex.

TL decreased with age. Scatter plots showing the participants’ TLs are shown in Fig 1.

TL was preprocessed using z-transformation to facilitate the comparison and achieve a standard normal distribution. This transformed value is the standardized TL (Eq. 3).


$$\begin{array}{c} \text{Standardized\textbackslash{}\_ TL }=\;(\text{TL }–\text{ mean(TL))/\textbackslash{} SD(TL)} \end{array}$$ (3)

**Fig 2:**
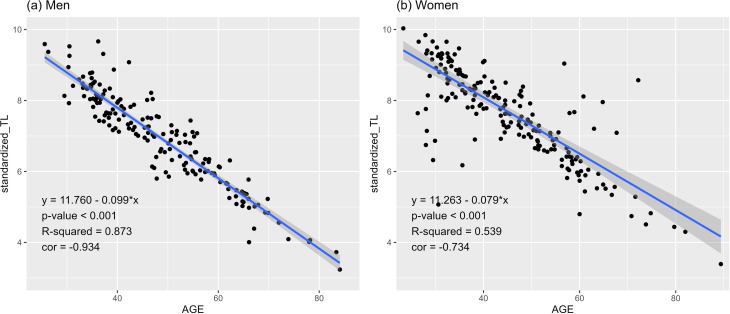
Correlation between age and standardized telomere length (standardized_TL) according to sex.

Scatter plots of standardized_TL and age revealed that standardized_TL provided a more effective explanation of the correlation with age than raw TL (Fig 2). Consequently, the preprocessed standardized_TL was applied as the final variable in the analysis. A strong negative correlation was observed between standardized_TL and age (men: −0.932, women: −0.734), whereby standardized_TL decreased significantly with increasing age (*p*-value < 0.001), indicating that TL decreased with increasing age.

*2) TMA modeling*

TMA was modeled using regression analysis with standardized_TL as the independent variable and age as the dependent variable (*p*-value < 0.001 in both sexes, R^2^ = 0.87 in men and 0.54 in women). The resulting TMA model can be expressed as follows (Eq. 4, 5).


$$\begin{array}{c} \text{TMA\textbackslash{} in\textbackslash{} men }=\;109.835\;-\;8.828\;\times \text{ standardized\textbackslash{}\_ TL} \end{array}$$ (4)

$$\begin{array}{c} \text{TMA\textbackslash{} in\textbackslash{} women }=\;97.427\;-\;6.790\;\times \text{ standardized\textbackslash{}\_ TL} \end{array}$$ (5)

**Fig 3:**
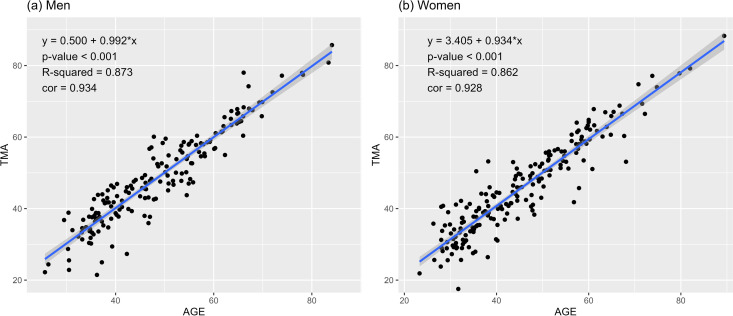
Correlation between age and telomere age (TMA).

A strong positive correlation was observed between TMA and age (men: 0.934, women: 0.928), whereby TMA increased with age (Fig 3).

3) Telomere age difference (TMAD)

TMAD was obtained by subtracting age from TMA and is an indicator of the difference between TMA and age, expressed as follows (Eq. 6).


$$\begin{array}{c} \text{TMAD }=\text{ TMA }–\text{ age} \end{array}$$ (6)

TMAD was used to examine differences between TMA and CA based on lifestyle-related factors.

### Statistical analysis

Regression analysis was used for TL estimation and TMA model development. Correlation and regression analyses were used to identify the correlation between TMA and lifestyle. All analyses were performed using R Studio (version 4.0.3), and the significance level was set at *p*-value ≤0.10.

## Results

### Characteristics of the participants

The study included 368 participants (174 men and 194 women) who met the established inclusion and exclusion criteria. Tables 1–3 present the mean ± standard deviation (SD) or percentage of the participants’ biomarkers and lifestyle-related parameters.

**Table 1:** Characteristics of the participants (mean ± SD): Age and biomarkers.

Biomarkers	Both sexes (*n* = 368)	Men (*n* = 174)	Women (*n* = 194)
Age (years)	46.82 ± 12.66	48.34 ± 12.34	45.44 ± 12.81
HT (cm)	164.92 ± 8.24	170.60 ± 6.78	159.80 ± 5.71
WT (kg)	65.28 ± 12.94	73.97 ± 11.53	57.45 ± 8.33
BF (kg)	19.01 ± 6.40	19.63 ± 6.57	18.46 ± 6.21
LBM (kg)	43.63 ± 9.22	51.34 ± 6.77	36.68 ± 4.29
WC (cm)	82.23 ± 11.21	89.05 ± 8.85	76.10 ± 9.44
HC (cm)	95.97 ± 7.08	98.53 ± 6.42	93.67 ± 6.88
SBP (mmHg)	121.54 ± 16.53	126.61 ± 12.16	116.98 ± 18.52
DBP (mmHg)	75.92 ± 11.65	79.33 ± 9.48	72.87 ± 12.55
FEV1 (mL)	2922.07 ± 716.97	3335.00 ± 606.27	2498.47 ± 557.69
FVC (mL)	3797.89 ± 912.87	4341.51 ± 748.78	3240.25 ± 707.37
TP (g/dL)	7.27 ± 0.42	7.26 ± 0.42	7.27 ± 0.42
ALB (g/dL)	4.41 ± 0.32	4.49 ± 0.30	4.33 ± 0.31
TB (mg/dL)	0.89 ± 0.46	0.98 ± 0.57	0.81 ± 0.31
AST (IU/L)	27.75 ± 16.40	31.84 ± 20.71	24.08 ± 9.91
ALT (IU/L)	27.13 ± 19.92	34.88 ± 23.67	20.18 ± 12.25
GGT (IU/L)	32.67 ± 35.15	46.24 ± 40.98	20.51 ± 23.01
TC (mg/dL)	201.46 ± 39.38	205.28 ± 43.56	198.02 ± 34.93
TG (mg/dL)	117.59 ± 85.44	141.38 ± 99.71	96.14 ± 63.11
HDL-C (mg/dL)	54.43 ± 13.74	48.37 ± 9.73	59.88 ± 15.55
LDL-C (mg/dL)	109.93 ± 26.94	115.27 ± 30.24	106.30 ± 23.93
HOMO (µmol/L)	8.75 ± 2.39	9.75 ± 2.28	7.48 ± 1.88
ALP (IU/L)	113.37 ± 69.33	115.03 ± 71.40	111.71 ± 67.37
LDH (IU/L)	285.98 ± 110.10	304.19 ± 107.63	268.57 ± 109.96
CPK (IU/L)	130.35 ± 196.60	168.63 ± 263.15	89.32 ± 53.27
FBS (mg/dL)	96.30 ± 19.45	100.72 ± 24.51	92.34 ± 12.16
HbA1c (%)	5.71 ± 1.22	5.79 ± 1.35	5.63 ± 1.06
BUN (mg/dL)	13.04 ± 3.42	13.82 ± 3.43	12.33 ± 3.27
CR (mg/dL)	0.76 ± 0.23	0.85 ± 0.17	0.69 ± 0.26
RBC (million cells/mcl)	4.69 ± 0.49	5.02 ± 0.39	4.37 ± 0.32
HEMO (Hb)	14.23 ± 1.71	15.52 ± 1.29	13.06 ± 1.11
HEMA (%)	42.30 ± 4.31	45.47 ± 3.17	39.37 ± 2.92

**Abbreviations:** HT, height; WT, weight; BF, body fat; LBM, lean body mass; WC, waist circumference; HC, hip circumference; SBP, systolic bold pressure; DBP, diastolic blood pressure; FEV1, forced expiratory volume in 1 s; FVC, forced vital capacity; TP, total protein; ALB, albumin; TB, total bilirubin; AST, aspartate aminotransferase; ALT, alanine aminotransferase; GGTP, gamma GTP; TC, total cholesterol; TG, triglyceride; HDL-C, high-density lipoprotein-cholesterol; LDL-C, low-density lipoprotein-cholesterol; HOMO, homocysteine; ALP, alkaline phosphatase; LDH, lactate dehydrogenase; CPK, creatine phosphokinase; FBS, fasting blood sugar; HbA1c, hemoglobin A1c; BUN, blood urea nitrogen; CR, creatinine; RBC, red blood cell; HEMO, hemoglobin; HEMA, hematocrit; SD, standard deviation.

**Table 2:** Characteristics of the participants (mean ± SD): Lifestyle (numeric).

Lifestyle	Both sexes (*n* = 368)	Men (*n* = 174)	Women (*n* = 194)
Number of weekly exercises	2.9 ± 2.0	3.1 ± 2.1	2.7 ± 1.9
Smoke1: number of cigarettes smoked per day	2.7 ± 5.9	5.0 ± 7.5	0.6 ± 2.5
Smoke2: total smoking years	4.1 ± 9.5	7.9 ± 12.3	0.7 ± 3.3
Alcohol consumption1: frequency of alcohol consumption per week	1.2 ± 1.6	1.7 ± 1.7	0.7 ± 1.2
Alcohol consumption 2: average number of glasses consumed per occasion	3.2 ± 4.9	4.7 ± 5.7	1.9 ± 3.6

**Abbreviations:** SD, standard deviation.

**Table 3:** Characteristics of the participants (%): Lifestyle (category).

Lifestyle	Both sexes (*n* = 368)	Men (*n* = 174)	Women (*n* = 194)
Exercise			
Never	104 (28.3%)	38 (21.8%)	66 (34.0%)
Once a week	86(23.4%)	43 (24.7%)	43 (22.2%)
Twice a week	50 (13.6%)	30 (17.2%)	20 (10.3%)
3 times a week	45 (12.2%)	16 (9.2%)	19 (14.9%)
4 times a week	26 (7.1%)	14 (8.0%)	12 (6.2%)
5 times a week	16 (4.3%)	8 (4.6%)	8 (4.1%)
6 times a week	18 (4.9%)	10 (5.7%)	8 (4.1%)
More than 7 times a week	15 (4.1%)	10 (5.7%)	5 (2.6%)
Sleep			
Less than 7 h	218 (59.4%)	102 (59.0%)	116 (59.8%)
7–8 h	140 (38.1%)	68 (39.3%)	72 (37.1%)
More than 8 h	9 (2.5%)	3 (1.7%)	6 (3.1%)
Stress			
Very severe	16 (4.4%)	13 (7.5%)	3 (1.5%)
Severe	71 (19.3%)	31 (17.9%)	40 (20.6%)
Normal	140 (38.1%)	61 (35.3%)	79 (40.7%)
Little (less)	109 (29.7%)	50 (28.9%)	59 (30.4%)
Very little (hardly)	30 (8.2%)	17 (9.8%)	13 (6.7%)

**Note:** STRESS: very severe (more than 70%), severe (50% ~ 70%), normal (30% ~ 50%), less (10% ~ 30%), hardly (less than 10%).

### Effects of lifestyle on TMAD

**Fig 4:**
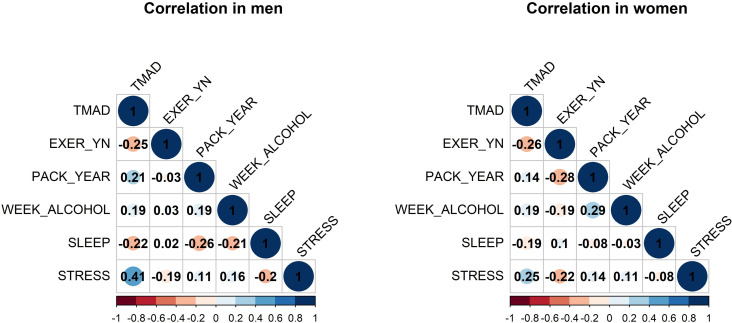
Correlation between telomere age difference (TMAD) and lifestyle.


Lifestyle-related parameters were used as independent variables, and TMAD was used as the dependent variable. The effects of lifestyle factors on TMAD were analyzed in men and women (Fig 4, Table 4). Exercise and sleep were negatively correlated with TMAD in both men and women, whereas the other factors exhibited a positive correlation (Fig 4). These findings are consistent with the regression coefficients presented in Table 4. The regression equations predicting TMAD according to sex can be expressed as follows (Eq. 7, 8).

**Table 4:** Multivariate associations of lifestyle-related parameters with TMAD.

Sex	Parameter	Estimate	Standardized	Std. error	t value	*p*-value
Men	(Intercept)	4.164		2.714	1.534	0.127
	EXER_YN	−7.808	−0.249	2.136	−3.655	< 0.001*
	PACK_YEAR	0.060	0.125	0.033	1.807	0.073*
	WEEK_ALCOHOL	0.367	0.103	0.246	1.492	0.138
	SLEEP	−0.864	−0.089	0.678	−1.275	0.204
	STRESS	1.429	0.320	0.313	4.565	< 0.001*
Women	(Intercept)	6.387		3.289	1.942	0.054*
	EXER_YN	−7.039	−0.179	2.830	−2.487	0.014*
	PACK_YEAR	0.045	0.015	0.226	0.201	0.841
	WEEK_ALCOHOL	0.991	0.125	0.561	1.767	0.079*
	SLEEP	−1.558	−0.155	0.682	−2.285	0.023*
	STRESS	1.006	0.185	0.377	2.672	0.008*

**Note:** R^2^ = 0.285; *p*-value < 0.001; EXER_YN: 0 (Never), 1 (others); SLEEP: 1 (others), 2 (7–8 h); STRESS: 1 (hardly), 2 (less), 3 (normal), 4 (severe), 5 (very severe); *: significant.

$$\begin{array}{c} \begin{array}{c} \text{Estimated\textbackslash{} TMAD\textbackslash{} in\textbackslash{} men}=4.164\;-\;7.808\;\times \;(\text{EXER\textbackslash{}\_ YN})\;+\;0.060\;\times \;(\text{PACK\textbackslash{}\_ YEAR})\;+\;0.367\;\\ \times \;(\text{WEEK\textbackslash{}\_ ALCOHOL})\;-\;0.864\;\times \;(\text{SLEEP})\;+\;1.429\;\times \;(\text{STRESS}) \end{array} \end{array}$$ (7)

$$\begin{array}{c} \begin{array}{c} \text{Estimated\textbackslash{} TMAD\textbackslash{} in\textbackslash{} women\textbackslash{} }=\;6.387\;-\;7.039\;\times \;(\text{EXER\textbackslash{}\_ YN})\;+\;0.045\;\times \;(\text{PACK\textbackslash{}\_ YEAR})\;\\ +\;0.991\;\times \;(\text{WEEK\textbackslash{}\_ ALCOHOL})\;-\;1.558\;\times \;(\text{SLEEP})\;+\;1.006\;\times \;(\text{STRESS}) \end{array} \end{array}$$ (8)

The estimated regression equations were significant for both men and women (*p*-value < 0.001). In men, the significant variables were exercise, smoking, and stress (*p*-value < 0.10), whereas in women, they were exercise, alcohol consumption, sleep, and stress (*p*-value < 0.10).

### Association between BA and TMA

**Fig 5:**
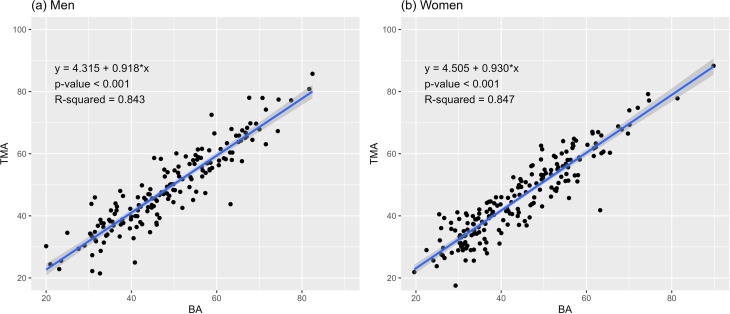
Association between biological age (BA) and telomere age (TMA).

Both TMA and BA are well-known indicators of an individual’s aging level. Scatter plots and regression analyses were used to examine the correlation between TMA and BA. The results of these analyses are presented in Table 5 and are visualized in Fig 5. The R^2^ values for both men and women indicated a high explanatory power (men: 0.843, women: 0.847), showing that an increase in BA by 1 year corresponded to a significant increase in TMA by approximately 1 year (men: 0.918 years, women: 0.930 years; *p*-value < 0.001).

**Table 5:** Association between biological age (BA) and telomere age (TMA).

Sex	Parameter	Estimate	Standardized	Std. Error	t value	*p*-value
Men	(Intercept)	4.315		1.544	2.795	0.006*
	BA	0.918	0.918	0.031	29.820	<0.001*
Women	(Intercept)	4.505		1.320	3.411	<0.001*
	BA	0.930	0.920	0.029	32.547	<0.001*

**Note:** R^2^ = 0.843; *p*-value < 0.001; *: significant.

## Discussion

This study investigated the correlation between TL and lifestyle-related parameters in 368 healthy Korean adults. Genomic DNA was extracted from blood samples, and TL was measured using MMQPCR. Lifestyle data were collected through surveys, and BA was measured using the MEDIAGE^TM^ system. The study aimed to comprehensively understand how lifestyle factors influence TMA, a TL-based variable.

Previous studies have explored the correlation between TL and lifestyle and dietary factors but have usually focused on one or at most two factors, such as smoking, alcohol consumption, or physical activity. In addition, a broad age range representing men and women have not been widely reported for a Korean population [51–55]. In this study, we modeled multiple lifestyle-related parameters to determine their correlation with TMA. Using multiple parameters in the model, our analysis yielded significant conclusions, with both men and women demonstrating similar results. The findings indicated that higher exercise frequency, reduced smoking and alcohol consumption, lower stress levels, and 7–8 h of sleep were associated with decreased TMA. A reduction in TMA suggests better health, as evidenced by the greater TLs in individuals of the same age and sex.

Kim et al. found that TL was significantly greater in individuals who engaged in habitual exercise than that in those with a more sedentary lifestyle (*p*-value < 0.01) [39]. In addition, the TL of smokers was shorter than that of non-smokers (*p*-value = 0.03) [40]. Epel et al. reported a negative correlation between TL and stress levels (R^2^ = –0.27) [7]. In particular, women experiencing elevated perceived stress had slightly shorter telomeres (95% confidence interval (CI)), which were also independently associated with their current smoking levels [8]. Schneider et al. found that both smoking and alcohol consumption were associated with shorter TLs [9]. Surtees et al. demonstrated that cigarette smoking was significantly associated with TL (*p*-value = 0.04, 95% CI = −0.010 to 0.036) [10]. Some studies have reported that high income, moderate physical activity levels, low BMI, smoking, and increased fruit and vegetable intake are positively associated with TL. Conversely, other studies have reported that psychological factors, such as stress or depression, shorter sleep duration, and smoking, as well as low education or income levels, are inversely related to TL [54,56–61]. Several studies have investigated the association between alcohol consumption and TL, and most have reported no significant association [54,62–64]; however, one study reported a significant association between alcoholism and TL [65].

Our study highlights the importance of maintaining a healthy lifestyle to help delay reductions in TL. Because genetic testing is excluded from basic health checkups, several people do not undergo TL testing. As a result, individuals who undergo genetic testing are more likely to receive personalized health services. Most lifestyle-related parameters tested in this study significantly affected TL; however, some variables exhibited only a trending effect.

## Conclusions

This study elucidated the complex relationship between lifestyle factors and TL in a cohort of healthy Korean adults. In both men and women, TMA decreased with increased exercise frequency, reduced smoking, alcohol consumption, stress levels, and 7–8 h of sleep. Consistent results across both sexes underscore the robustness of these findings. These results emphasize the significance of adopting a healthy lifestyle to delay telomere shortening and promote overall health and effective aging management. Future large-scale investigations are required to obtain results with greater statistical power. The quantified values presented in this study can help motivate individuals to proactively manage their lifestyles to delay aging and maintain optimal health. This study provides a foundation for future research to elucidate the intricate interplay between lifestyle and TL.
